# Analysis of alcohol-metabolizing enzymes genetic variants and RAR/RXR expression in patients diagnosed with fetal alcohol syndrome: a case-control study

**DOI:** 10.1186/s12864-024-10516-7

**Published:** 2024-06-17

**Authors:** Melina Vieiros, Elisabet Navarro-Tapia, Anna Ramos-Triguero, Àgueda García-Meseguer, Leopoldo Martínez, Óscar García-Algar, Vicente Andreu-Fernández

**Affiliations:** 1 Grup de Recerca Infància i Entorn (GRIE), Institut d’investigacions Biomèdiques August Pi i Sunyer (IDIBAPS), Barcelona, Spain; 2 IdiPAZ - Instituto de Investigación Hospital Universitario La Paz, Madrid, Spain; 3 Department de Cirurgia i Especialitats Mèdico-Quirúrgiques, Universitat de Barcelona, Barcelona, Spain; 4 Faculty of Health Sciences, Valencian International University, Valencia, Spain; 5 Department of Pediatric Surgery, Hospital Universitario La Paz, Madrid, Spain; 6 Department of Neonatology, Hospital Clínic-Maternitat, ICGON, BCNatal, Barcelona, Spain; 7 Biosanitary Research Institute, Valencian International University, Valencia, Spain

**Keywords:** Alcohol, Fetal alcohol syndrome, FASD, Retinoic acid, ADH, Retinoid X receptor, Retinoic acid receptor, ALDH, Polymorphisms, Expression

## Abstract

Understanding the mechanisms underlying alcohol metabolism and its regulation, including the effect of polymorphisms in alcohol-metabolizing enzymes, is crucial for research on Fetal Alcohol Spectrum Disorders. The aim of this study was to identify specific single nucleotide polymorphisms in key alcohol-metabolizing enzymes in a cohort of 71 children, including children with fetal alcohol syndrome, children prenatally exposed to ethanol but without fetal alcohol spectrum disorder, and controls. We hypothesized that certain genetic variants related to alcohol metabolism may be fixed in these populations, giving them a particular alcohol metabolism profile. In addition, the difference in certain isoforms of these enzymes determines their affinity for alcohol, which also affects the metabolism of retinoic acid, which is key to the proper development of the central nervous system. Our results showed that children prenatally exposed to ethanol without fetal alcohol spectrum disorder traits had a higher frequency of the ADH1B*3 and ADH1C*1 alleles, which are associated with increased alcohol metabolism and therefore a protective factor against circulating alcohol in the fetus after maternal drinking, compared to FAS children who had an allele with a lower affinity for alcohol. This study also revealed the presence of an ADH4 variant in the FAS population that binds weakly to the teratogen, allowing increased circulation of the toxic agent and direct induction of developmental abnormalities in the fetus. However, both groups showed dysregulation in the expression of genes related to the retinoic acid pathway, such as retinoic acid receptor and retinoid X receptor, which are involved in the development, regeneration, and maintenance of the nervous system. These findings highlight the importance of understanding the interplay between alcohol metabolism, the retinoic acid pathway and genetic factors in the development of fetal alcohol syndrome.

**Supplementary Information:**

The online version contains supplementary material available at 10.1186/s12864-024-10516-7.

## Background

Alcohol is a teratogen and its consumption during pregnancy can produce severe effects on fetal development. Due to its physicochemical properties (polar and small size), it can freely cross the placental barrier and be distributed in fetal tissues [1]. In Europe, recent studies concluded that 25.2% f pregnant women consume alcohol occasionally or chronically at some time during pregnancy [2]. The severity of the alcohol damage depends on maternal and fetal genetic factors, the dose of alcohol consumed, the stage of pregnancy, nutritional status, and drinking patterns [3]. The fetal brain is particularly vulnerable to alcohol because of its gradual vascularization during pregnancy and the absence or reduced expression of some alcohol-metabolizing enzymes [4].

The full range of clinical manifestations associated with prenatal alcohol exposure (PAE) is encompassed by Fetal Alcohol Spectrum Disorders (FASD) [5], with four diagnostic categories according to the Institute of Medicine (IOM) diagnostic criteria: Fetal Alcohol Syndrome (FAS), Partial Fetal Alcohol Syndrome (pFAS), Alcohol-Related Neurodevelopmental Disorder (ARND) and Alcohol-Related Birth Defects (ARBD) [6]. FAS is the most severe manifestation and is characterised by pre- and postnatal growth retardation, morphological malformations, cranioencephalic defects with a characteristic facial pattern (short palpebral fissures, smooth philtrum, and thinner upper lip) [7, 8], and problems in the development of the central nervous system (CNS). Deficits in brain growth are associated with cognitive problems (including poor memory, inability to understand concepts, poor comprehension of language, poor problem-solving skills, among others) and behavioural problems (including hyperactivity, inability to concentrate, impulsivity and anxiety) [9, 10]. The global prevalence of FASD is approximately 7.7 per 1000 births (95% CI = 4.9–11.7) [11]. However, the wide range of symptoms, the social stigma associated with alcohol dependence and the difficulty of early diagnosis have led to an underestimation of the data.

The enzymes responsible for metabolising alcohol in the fetus once it has crosses the placenta are the same as those in the mother, although their activity and concentration are different. Ethanol oxidation is catalysed by the cytosolic enzyme alcohol dehydrogenase (ADH), which produce acetaldehyde. Acetaldehyde causes oxidative damage and depletes glutathione levels, leading to cell death [12]. Acetaldehyde is then metabolized by aldehyde dehydrogenase (ALDH) to produce acetic acid. Ethanol can also be metabolized by cytochrome P450 2E1 (CYP2E1), the major ethanol-inducible P450 isoenzyme, which is expressed mainly in the liver and to a lesser extent in the brain, at high doses (> 240 g per day) or after chronic exposure to ethanol [13]. In the case of placental CYP2E1, its affinity for ethanol is higher than placental ADH and its metabolism produces radical oxygen species (ROS), promoting cellular damage and apoptosis [14]. As a result, the increased toxicity in cells leads to neurodevelopmental damage characteristic of FASD patients [15]. Regarding fetal metabolism, once ethanol has entered the fetal circulation, the fetus attempts to metabolize it through the same pathways seen above, although the metabolic capacity in the fetus is much lower than in the adult and CYP2E1 and ADH are active from 16 to 26 weeks of gestation, respectively [16].

ADH enzymes are encoded by seven genes and are divided into five classes, of which classes I and II are the most important for ethanol metabolism. Class I is controlled by three gene loci, ADH1, ADH2 and ADH3 (also known as ADH1A, ADH1B and ADH1C, respectively) and has a high affinity for ethanol, contributing to acetaldehyde synthesis. Class II is encoded by ADH4, which is mainly found in the liver and has a high Km for ethanol, requiring a higher substrate concentration to reach 50% Vmax [17]. However, ADH4 is less efficient for alcohol catabolism than other isoforms expressed in adults [18]. ALDH enzymes are encoded by several genes and are divided into different classes, with class 1 which is regulated by multiple gene loci (ALDH1A1, ALDH1A2 and ALDH1A3) and class 2 being the most relevant in acetaldehyde metabolism [19]. Class 2, represented by ALDH2, is of particular importance in the detoxification of acetaldehyde generated during ethanol metabolism in the liver [20]. Nevertheless, certain genetic variants of ALDH2 may result in reduced enzyme activity, which may influence alcohol tolerance and increase the risk of adverse side effects [20].

The study the single nucleotide polymorphisms (SNPs) in ADH, ALDH, and CYP2E1 is relevant to FASD because it can alter the catalytic function of these hepatic, neuronal, and fetal enzymes, leading to changes in the levels of ethanol and its toxic metabolites [21]. A summary of the published work on these SNPs is shown in Tables 1 and 2. In addition, the presence of certain SNPs may act as a protective factor against alcohol abuse and other alcohol-related diseases [22]. For example, the ADH1B*1 predominates in Caucasian populations. Chinese, Japanese and Korean individuals mainly have ADH1B*2, and the ADH1B*3 allele has been found in African-American populations. ALDH2*2, the less active form of the enzyme, is more common in Asian populations [23]. Therefore, a combination of a high metabolism activity (ADH1B*2 and ADH1B*3) and the inactive form ALDH2*2 will be associated with less alcohol dependence [24] due to the “flushing” effect, an unpleasant phenomenon after alcohol consumption.

**Table 1:** ADH1B and ADH1C SNPs described in the bibliography which modulate the activity of the enzymes that they codify

Enzyme	SNP	Position	Allele variation	Aminoacid variation	Allele	Subunit	Amino acid differences in alleles	Turnover rate (min^− 1^)
ADH1B	rs1229984 rs2066702	Exon 3 Exon 9	G/A C/T	Arg48His Arg370Cys	ADH1B*1 (C)	β_1_	Arg48, Arg370	4
					ADH1B*2 (A)	β_2_	His48, Arg370	350
					ADH1B*3 (T)	β_3_	Arg48, Cys370	300
ADH1C	rs698 rs1693482	Exon 8 Exon 6	G/A C/T	Ile350Val Arg272Gln	ADH1C*1 (C)	γ_1_	Arg272, Ile350	90
					ADH1C*2 (T)	γ_2_	Gln272, Val350	40

The activity of some enzymes depends on two polymorphisms. Turnover rate indicates how many molecules of ethanol the enzyme converts to acetaldehyde per minute at saturating ethanol concentrations. For more details see [23, 25, 26]

**Table 2:** ADH4, CYP2E1 and ALDH2 SNPs described in the bibliography which modulate the activity of the enzymes that they codify

Enzyme	SNP	Position	Allele variation	Aminoacid variation	Allele	Subunit	Amino acid differences in alleles	Activity
ADH4	rs1800759	Promoter region at 5′ end	A/C	–	–	–	–	Half the promoter activity
CYP2E1	rs72559710	Exon 2	G/A	Arg76His	CYP2E1*2	–	His76	Reduce percentage of expression
	rs55897648	Exon 8	A/G	Ile389Val	CYP2E1*3	–	Val389	
	rs6413419	Exon 4	G/A	Val179Ile	CYP2E1*4	–	Ile179	
	rs3813867	5’- flanking region	G/C	–	CYP2E1*5B or c2	–	–	Increment up to 10- times
	rs2031920		C/T					
	rs6413432	Intron 6	T/A	–	CYP2E1*6	–	–	Up-regulated transcription
ALDH2	rs671	Exon 12	G/A	Glu487Lys	ALDH2*1	–	Glu487	Active
					ALDH2*2	–	Lys487	Inactive

For more details see [23, 25, 26]

Retinoids are a group of compounds derived from vitamin A, such as retinol and retinoic acid (RA). These compounds play a significant role in modulating the expression of enzymes involved in ethanol metabolism, such as the CYP2E1, ADH, and ALDH families [27]. RA also participates in the regulation of vertebrate embryogenesis, by modulating gene expression in the nucleus after binding to the retinoic acid receptor (RAR) and retinoid X receptor (RXR) transcription factors. Both RAR and RXR are composed of three proteins (α, β and γ). Once RXR is homo- or heterodimerized with RAR, they bind to RA response elements (RAREs) or retinoid X response elements (RXRE), located in the promoter sequence of target genes, leading to changes in differentiation, growth, and homeostasis processes [28].

A lack of Vitamin A during pregnancy can lead to developmental malformations in the fetus [29–31]. Ethanol has been linked to disruptions in retinoid signalling, which may contribute to harmful effects on embryonic development. Ethanol can interfere with retinol metabolism, leading to changes in RA synthesis and, consequently, changes in the gene expression of RAR and RXR [32]. Ethanol and retinol metabolism involve the same enzyme families. In adults, the first enzymatic step is based on the ADH oxidation and the generation of acetaldehyde and retinaldehyde, respectively. The second oxidation is produced by ALDH and the retinaldehyde dehydrogenase (RALDH), resulting in acetic acid and retinoic acid. In the fetus, the first oxidation of retinol is performed mainly by members of the short-chain dehydrogenase/reductase family, such as RDH10 [33] and its product, the retinaldehyde, competes for RALDH2 in the presence of acetaldehyde, inhibiting retinoic acid biosynthesis [34]. Recent studies in *Xenopus laevis* have shown that ethanol and its oxidation product, acetaldehyde, repress RA signalling in a similar way during early embryogenesis [34]. Furthermore, acetaldehyde competes with retinaldehyde for the available RALDH2 activity, which catalyses the synthesis of RA from retinaldehyde [34]. Therefore, ethanol consumption leads to a reduction in RA synthesis and signalling [35–37]. RA is also metabolized by CYP2E1 after a long-term and excessive ethanol intake, causing a decrease in RA levels in plasma and liver [38].

Previous genetic studies have focused on the role of enzymes related to alcohol metabolism in liver disease in the adult population [39]. However, there is a scarcity of studies on genetic variability in key alcohol metabolism enzymes in children with FAS. This study analysed the allelic and genotypic frequencies of relevant SNPs in the main ethanol-metabolising enzymes in a Caucasian control and FAS population, as well as in children with prenatal alcohol exposure (PAE) with no FASD diagnosis. Thus, the aim of this study was to investigate a possible positive selection on certain genetic polymorphisms. Additionally, differences in the gene expression of *RAR* and *RXR* were also compared among groups to find affectations in the RA pathway due to prenatal ethanol exposure.

## Methods

### Patients

This study was conducted in a cohort of 71 children (27 girls and 44 boys) aged 7 to 15 years. 34 children were diagnosed with FAS and 37 without FASD, 9 of them with confirmed PAE. The children were adopted from Eastern European countries without any known genetic condition and prenatal alcohol exposure was confirmed for all children by adoption medical reports or adoption court rulings. A similar confirmation with a negative history of drug use by interviewing the mother was obtained for the 28 children without FASD who were considered as the control population [40]. The study protocol was approved by the local ethics committee (CEIC-PSMAR: Comitè Ètic d’Investigació Clínica- Parc de Salut Mar) (2013/5272/I) and was carried out at the Institut Hospital del Mar d’Investigacions Mèdiques (IMIM). All children recruited in this study, FAS, PAE and control children, were independently evaluated by FAS dysmorphologists according to the standardized dysmorphology exams for FASD diagnosis [41], and their classification in the FAS group was made according to the clarification of the 1996 IOM criteria, revised in 2005 [5]. Specifically, paediatricians with clinical experience in the diagnosis of FASD. Full details of the clinical assessment and diagnosis of these children, as well as their recruitment, can be found in a previous study [42].

### SNPs determination

#### Sample collection and DNA extraction

After a clinical evaluation and a neurocognitive assessment for the previous study [42], a blood sample was collected and processed immediately. DNA was extracted from 200 µL of whole blood using the Purelink Genomic DNA Mini Kit (K1820-01, Thermo Fisher Scientific) according to the manufacturer’s recommendations. The quantity and quality of the extracted DNA was then checked using Nanodrop (Thermo Fisher Scientific, Nanodrop 1000). Samples were identified with a letter and number code and immediately stored at -80 °C until further analysis.

#### Primer designing and tetra-ARMS assay

Genomic DNA extracted from blood was used for Tetra-ARMS PCR amplification to identify the allelic variants of 13 SNPs of the following alcohol metabolism enzymes: ADH1B, ADH1C, ADH4, ALDH1A1 and CYP2E1.

The PCR-Tetra-ARMS uses 4 primers to determine the genotype and allelic variants in heterozygous or homozygous states. The two outer primers amplify a region of the gene containing the genotype to be analysed, while the two inner primers are designed with the last nucleotide of each primer in the 3’ direction of polymerase synthesis complementary to each of the allelic variants of the SNP being studied. In this way, a large fragment of the target gene and smaller allele-specific amplicons can be resolved by electrophoresis [43].

Primers for tetra-ARMs PCR were designed using the Gene Runner programme (http://generunner.com), with a length between 18–25 bp and the nucleotide of the 3’ of the inner primers containing a mismatch according to the SNPs studied. The annealing temperature for each group of primers was evaluated by gradient PCR, using a range of temperatures from 50°C to 65°C. The specificity of all the primers was tested to develop the optimal conditions for the reaction. The primers sequence and amplification conditions are detailed in Additional file 1.

PCR reactions were performed using GoTaq® G2 Flexi DNA Polymerase (Promega Biotech Iberica S.L., M7801) with the following conditions: 10 min of initial DNA denaturation at 95°C, 40 cycles of denaturation at 95°C for 40s, annealing at tested temperatures for 1 min and an extension step at 72°C for 1 min, with a final extension at 72°C for 10 min. The amplified products (10µL for each sample) were stained with SYBR Safe (S33102, ThermoFisher Scientific) and resolved on 1.5% agarose gel electrophoresis to determine the SNPs. Those SNPs that could not be studied due to the impossibility of obtaining specific primers compatible with the tetra-ARMS methodology (7 in total) were analysed by Sanger sequencing (Additional file 2). PCR conditions were the same as above and the amplification products were purified according to the manufacturer’s protocol of the FavorPrep GEL/PCR Purification Kit (FAGCK001, Favorgen Biotech Corporation). The quantity and quality of the purified DNA was checked using Nanodrop (Thermo Fisher Scientific, Nanodrop 1000) and the amplified products were sent to NZYTECH company (Portugal) for sequencing.

### Gene expression analysis

#### RNA extraction

The gene expression of *RAR* and *RXR* was assessed by quantitative PCR (RT-qPCR). RNA was obtained from 10 control patients, 10 FAS patients and 8 PAE patients from the total number of patients included in this study. Briefly, 4 mL of blood collected in BD Vacutainer K3E Aprotinin tubes (Ref: 361,017, BD Biosciences) was transferred to a BD Vacutainer® CPT™ tube (Ref: 362,760, BD Biosciences), inverted several times and centrifuged (25 min at 1750 g). Peripheral blood mononuclear cells (PBMCs) were isolated by density gradient centrifugation. After washing with PBS, cells were homogenised using the QIAshredder (Qiagen, Clayton, Australia) and RNA isolation from the homogenised lysate was performed using the RNeasy MiniKit (Qiagen) according to the manufacturer’s instructions. RNA quantity and quality were measured using Nanodrop (Thermo Fisher Scientific, Nanodrop 1000) and immediately frozen by immersion in liquid nitrogen. RNA samples were stored at −80 °C for further analysis.

#### RT-qPCR

For RT-qPCR analysis, 100 ng of RNA was retrotranscribed into cDNA using a high-capacity reverse transcription kit according to the manufacturer’s protocol (4368814, Applied Biosystems). Primer design for all *RAR* and *RXR* genes tested was performed using the Primer Blast program (https://www.ncbi.nlm.nih.gov/tools/primer-blast/) with to the following conditions: amplicons between 100 and 300 bp, melting temperature of 58–60°C and non-specific hybridisation (Additional file 3). To evaluate the specificity and efficiency of each pair of primers, standard curves were generated and those primers with 90–110% efficiency were selected.

RT-qPCR was performed in triplicate for each sample and 10µL of the Master Mix was prepared with the following composition: 1µL of cDNA (100ng), 3µL of nuclease-free water, 5µL of SYBR Green Reagent (95074-012, QuantaBio), 0.5µL of forward primer (5µM) and 0.5µL of reverse primer (5µM). RT-qPCR was performed in QuantStudioTM 7 Real-Time PCR (Thermo Fisher Scientific) with the following conditions: 20 s at 95°C to denature the cDNA, 40 cycles of 1 s denaturation at 95°C, alignment of the sequences for 1 min at 60°C and extension of the reaction for 15 s at 95°C, leaving 1 min at 60°C to complete amplification of all sequences. Finally, the relative expression of each gene was normalised to the housekeeping gene, GAPDH, and measured by 2-ΔΔCT method [44].

### Hardy-Weinberg and statistical analyses

Our study analysed several SNPs related to alcohol metabolism enzymes using a chi-squared test (χ²) to compare allele and genotypic frequencies in different cohorts (FAS, control, and PAE). The analysis also assessed whether the population samples adhered to Hardy-Weinberg equilibrium (HWE), a principle that states that genetic diversity within a population remains consistent across generations in the absence of external influences. In populations where mating occurs randomly and there are no external disturbances, the Hardy-Weinberg principle states that both genotype and allele frequencies will remain stable, thus achieving equilibrium.

To determine Hardy-Weinberg equilibrium, the observed distribution of genotypes within the population is used to calculate the expected frequencies of dominant and recessive alleles. The calculated frequencies were compared with the expected genotypic frequencies in a Hardy-Weinberg Caucasian population. For each superpopulation, the ratio of observed to expected (O/E) heterozygous carriers, assuming Hardy-Weinberg equilibrium, was computed and assessed using chi-squared testing.

One-way analysis of variance (ANOVA) in Graphpad Prism8 software was used to compare the relative gene expression of *RAR* and *RXR* by RT-qPCR in blood samples from control, FAS, and PAE patients. Statistical significance was set at a p-value of less than 0.05.

## Results

**Fig. 1:**
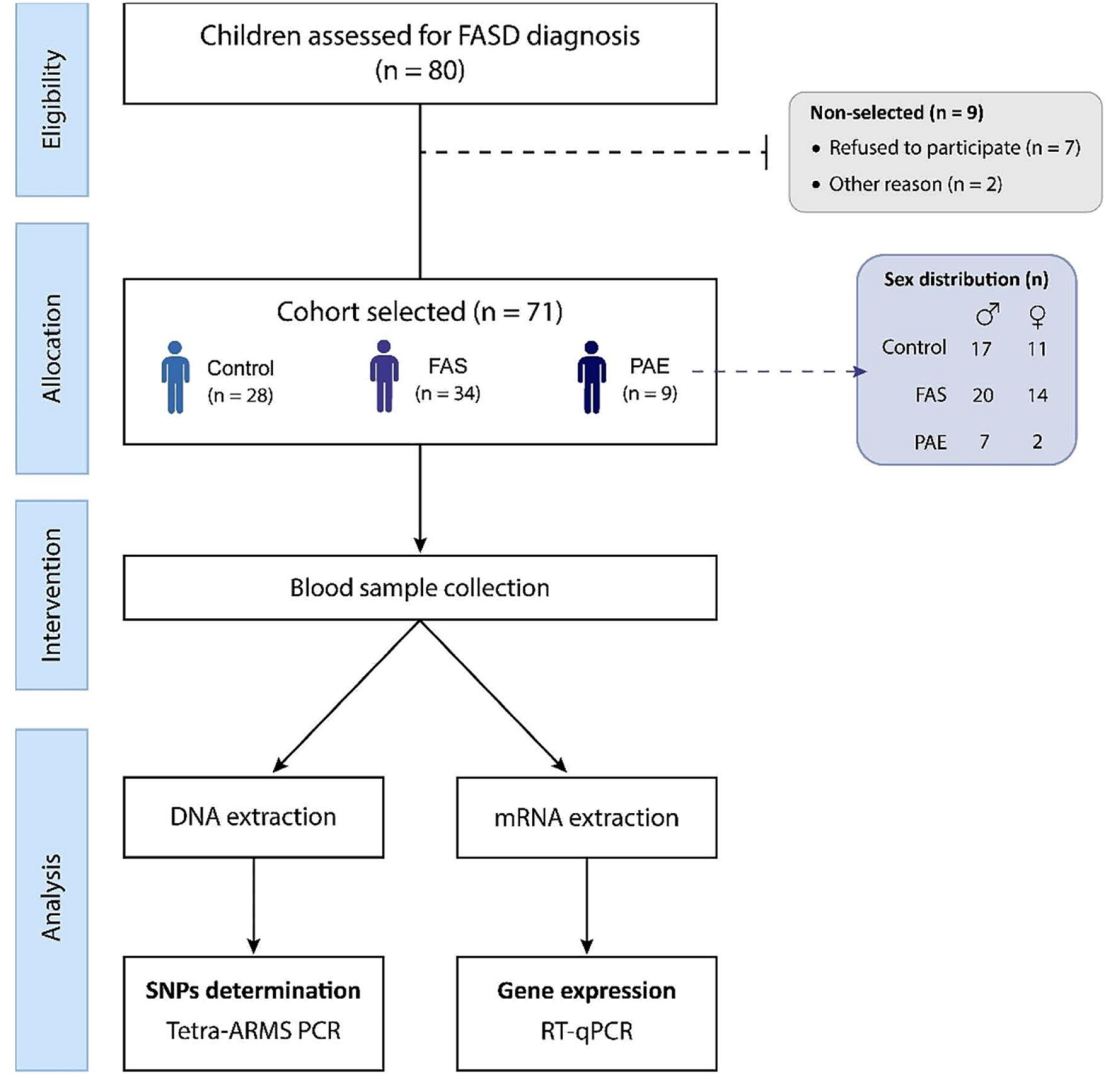
Workflow diagram of the study

The current study initially included 80 children adopted from Eastern European countries (EEC), of whom 9 were not selected for the following reasons: 7 refused to participate and 2 for other private reasons. The remaining 71 patients who participated in this study were aged between 7 and 15 years and were divided into 3 groups, FAS (*n* = 34); PAE (*n* = 9) and 28 control children (Fig. 1). There were no significant differences in age, sex or pre-existing comorbidities between FAS, PAE and control patients. Blood samples were collected for DNA and mRNA extraction, SNPs and gene expression analysis.

### Determination of alcohol metabolism enzyme SNPs

#### ADH1B

In ADH1B, the allelic and genotypic frequencies of each patient were calculated for the SNPs rs1229984 and rs2066702 (Table 3). SNP rs1229984 changes guanine to adenine, which causes an arginine to histidine modification at protein position 48. The presence of guanine correlates with the ADH1B*1 variant, the less active form of the protein. By contrast, adenine determines the ADH1B*2 polymorphism, a protective allele against heavy alcohol consumption [45]. Our results showed no statistical significance for this SNP between the groups studied. Regarding to ADH1B*3, a significant increase in the frequency of the T allele (11%) was observed (p-value = 0.04) in the PAE group compared to the FAS group (1%) [46], whose genotype (CC) was more frequent compared to PAE (97% vs. 77%).

**Table 3:** Genotype and allele frequencies of the ADH1B SNPs rs1229984 and rs2066702 in control, FAS and PAE children

ADH1B SNPs			Control		FAS		PAE		Statistical hypothesis testing		
			n	Freq	n	Freq	n	Freq	Groups	χ2	*p*-value
rs1229984 (G →A)	Genotype	AA	1	0.03	3	0.09	0	0	Ctrl vs. FAS	2.10	0.35
		AG	3	0.11	1	0.03	1	0.11	Ctrl vs. PAE	0.33	0.84
		GG	24	0.86	30	0.88	8	0.89	PAE vs. FAS	1.81	0.40
	Allele	A	5	0.08	7	0.10	1	0.06	Ctrl vs. FAS	0.07	0.79
		G	51	0.91	61	0.90	17	0.94	Ctrl vs. PAE	0.21	0.66
									PAE vs. FAS	0.38	0.54
rs2066702 (C → T)	Genotype	CC	24	0.86	33	0.97	7	0.77	Ctrl vs. FAS	2.66	0.26
		CT	4	0.14	1	0.03	2	0.22	Ctrl vs. PAE	0.31	0.85
		TT	0	0	0	0	0	0	PAE vs. FAS	4.08	0.13
	Allele	C	52	0.93	67	0.99	16	0.89	Ctrl vs. FAS	2.55	0.11
		T	4	0.07	1	0.01	2	0.11	Ctrl vs. PAE	0.28	0.59
									PAE vs. FAS	3.93	0.04*

*Abbreviations* FAS: Fetal Alcohol Syndrome; PAE: Prenatal Alcohol Exposure; rs1229984 allele A: ADH1B*2; rs2066702 allele C: ADH1B*1; rs2066702 allele T: ADH1B*3. * Indicates statistical significance (*p*-value < 0.05). χ2: Chi square test

#### ADH1C

Rs698 is a polymorphism of ADH1C in which an adenine is replaced by a guanine, defining two different variants: ADH1C*1 with the G allele and ADH1C*2 with the A allele, with different affinity for ethanol. The genetic variant ADH1C*2 would be more common in slow metabolisers of alcohol and would therefore have a longer duration of ethanol presence and a lower amount of acetaldehyde in the blood. Although the results were not significant (Table 4), we observed a higher frequency of the GG genotype in the PAE group (33%) compared with the control group (4%). This ADH1C*1 isoform, with 2.5 times higher rate of alcohol metabolism, could produce a flushing effect due to higher levels of acetaldehyde in the blood and lead to an aversion to alcohol.

Regarding the rs1693482 polymorphism, the control group had a significantly higher frequency of CT heterozygotes (92%) than the PAE group (56%). In the PAE group, the CC genotype was higher (33%) than the control group (4%), confirming the higher proportion of ADH1C*1 in the prenatally exposed group, as seen above. No statistical differences were found between FAS and the other groups.

**Table 4:** Genotype and allele frequencies of ADH1C (γ) for rs698 and rs1693482 in control, FAS and PAE children

ADH1C (γ) SNPs			Control		FAS		PAE		Statistical hypothesis testing		
			n	Freq	n	Freq	n	Freq	Groups	χ2	*p*-value
rs698 (A →G)	Genotype	AA	9	0.32	11	0.32	2	0.22	Ctrl vs. FAS	0.55	0.76
		AG	18	0.64	17	0.5	4	0.45	Ctrl vs. PAE	0.68	0.71
		GG	1	0.04	6	0.18	3	0.33	PAE vs. FAS	0.28	0.867
	Allele	A	36	0.64	39	0.57	8	0.44	Ctrl vs. FAS	0.62	0.432
		G	20	0.36	29	0.43	10	0.56	Ctrl vs. PAE	2.22	0.14
									PAE vs. FAS	0.96	0.33
rs1693482 (C → T)	Genotype	CC	1	0.04	6	0.18	3	0.33	Ctrl vs. FAS	3.19	0.20
		CT	26	0.92	26	0.76	5	0.56	Ctrl vs. PAE	6.71	0.03*
		TT	1	0.04	2	0.06	1	0.11	PAE vs. FAS	1.27	0.53
	Allele	C	28	0.50	38	0.56	11	0.61	Ctrl vs. FAS	0.43	0.51
		T	28	0.50	30	0.44	7	0.39	Ctrl vs. PAE	0.67	0.41
									PAE vs. FAS	0.16	0.69

*Abbreviations* FAS: Fetal Alcohol Syndrome; PAE: Prenatal Alcohol Exposure; rs698 allele A: ADH1C*1; rs698 allele G: ADH1C*2; rs1693482 allele C: ADH1C*1; rs1693482 allele T: ADH1C*2. * Indicates statistical significance (*p*-value < 0.05). χ2: Chi square test

#### ADH4

The SNPs rs1042364 and rs1800759 were analyzed by tetra-Arms PCR assay, whereas first generation sequencing was used for rs1126671 and rs29001219. Ethanol has a higher affinity for ADH4 than retinol [47] so the competition between these molecules for the active site of ADH4 triggers a reduction in RA synthesis [47].

The results from ADH4 showed no allelic and genotypic statistical differences between groups, except for rs1126671 (Table 5). A significant increase in GG homozygotes in the group diagnosed with FAS (62%) compared to the control group (39%) was observed (*p*-value = 0.004).

**Table 5:** Genotype and allele frequencies of ADH4 (π) for rs1126673, rs1042364, rs1800759, rs1126671 and rs29001219 in control, FAS and PAE children

ADH4 (π) SNPs			Control		FAS		PAE		Statistical hypothesis testing		
			n	Freq	n	Freq	n	Freq	Groups	χ2	*p*-value
rs1126673 (A → G)	Genotype	AA	7	0.25	7	0.21	1	0.11	Ctrl vs. FAS	0.13	0.93
		AG	21	0.75	25	0.73	8	0.89	Ctrl vs. PAE	0.77	0.67
		GG	0	0	2	0.06	0	0	PAE vs. FAS	0.56	0.75
	Allele	A	35	0.62	39	0.57	10	0.56	Ctrl vs. FAS	0.33	0.56
		G	21	0.38	29	0.43	8	0.44	Ctrl vs. PAE	0.27	0.59
									PAE vs. FAS	0.02	0.89
rs1042364 (A → G)	Genotype	GG	20	0.71	28	0.82	8	0.89	Ctrl vs. FAS	2.47	0.29
		AG	8	0.29	5	0.15	1	0.11	Ctrl vs. PAE	1.13	0.57
		AA	0	0	1	0.03	0	0	PAE vs. FAS	0.37	0.83
	Allele	G	48	0.86	61	0.9	17	0.94	Ctrl vs. FAS	0.46	0.50
		A	8	0.14	7	0.1	1	0.06	Ctrl vs. PAE	0.97	0.32
									PAE vs. FAS	0.39	0.54
rs1800759 (A → C)	Genotype	AA	2	0.07	3	0.09	0	0	Ctrl vs. FAS	3.43	0.18
		AC	21	0.75	18	0.53	7	0.78	Ctrl vs. PAE	0.71	0.70
		CC	5	0.18	13	0.38	2	0.22	PAE vs. FAS	2.07	0.35
	Allele	A	25	0.45	24	0.35	7	0.39	Ctrl vs. FAS	1.13	0.19
		C	31	0.55	44	0.65	11	0.61	Ctrl vs. PAE	1.18	0.67
									PAE vs. FAS	0.08	0.78
rs1126671 (A → G)	Genotype	GG	11	0.39	21	0.62	6	0.66	Ctrl vs. FAS	10.89	0.004*
		AG	17	0.61	8	0.23	3	0.33	Ctrl vs. PAE	1.50	0.47
		AA	0	0	5	0.15	0	0	PAE vs. FAS	1.10	0.58
	Allele	G	39	0.70	50	0.74	15	0.83	Ctrl vs. FAS	0.23	0.63
		A	17	0.30	18	0.26	3	0.17	Ctrl vs. PAE	0.92	0.34
									PAE vs. FAS	0.52	0.47
rs29001219 (A → G)	Genotype	GG	28	1	34	1	9	1	Ctrl vs. FAS	0	1
		AG	0	0	0	0	0	0	Ctrl vs. PAE	0	1
		AA	0	0	0	0	0	0	PAE vs. FAS	0	1
	Allele	G	56	1	68	1	18	1	Ctrl vs. FAS	0	1
		A	0	0	0	0	0	0	Ctrl vs. PAE	0	1
									PAE vs. FAS	0	1

*Abbreviations* FAS: Fetal Alcohol Syndrome; PAE: Prenatal Alcohol Exposure. * Indicates statistical significance (*p*-value < 0.05). χ2: Chi square test

#### ALDH1A1, ALDH2 and CYP2E1

Three SNPs of the ALDH1A1 enzyme were evaluated: rs1049981, rs11554423 and rs8187929, as well as rs671 and rs769724893 of ALDH2. Our results showed that the ancestral allele for all polymorphisms evaluated was detected in all subjects studied, with no differences between groups (Additional file 4). No differences were also found in the SNPs studied for ALDH2, where the GG genotype appeared in 100% of cases in all three groups.

We also observed no differences in ALDH2 genotype between groups, considering the importance of this gene in the protection against alcoholism, we decided to analyze the expression of the enzyme in all groups. As a first approximation, with few patients and a semi-quantitative analysis by RT-PCR, the results showed a significantly higher expression of ALDH2 enzyme in the FAS population, compared to the control group and PAE (Additional file 5). Also, the PAE group had a significantly lower expression of the enzyme than the FAS and control population.

Regarding CYP2E1, we tested six SNPs (rs72559710, rs55897648, rs6413419, rs2031920, rs3813867 and rs6413432) to detect gene isoforms specifically associated with the selected groups. However, there were no statistically significant differences between the allelic and genotypic frequencies of the study groups (Additional file 4).

### Retinoic acid metabolism

The mRNA levels of the main subtypes of RAR and RXR (*RARα1*, *RARα2*, *RARβ1*, *RARβ2*, *RARγ*, *RXRα1*, *RXRα2*, *RXRβ1*, *RXRβ2* and *RXRγ*) were examined to determine if there was any change in gene expression due to alcohol exposure during pregnancy.

**Fig. 2:**
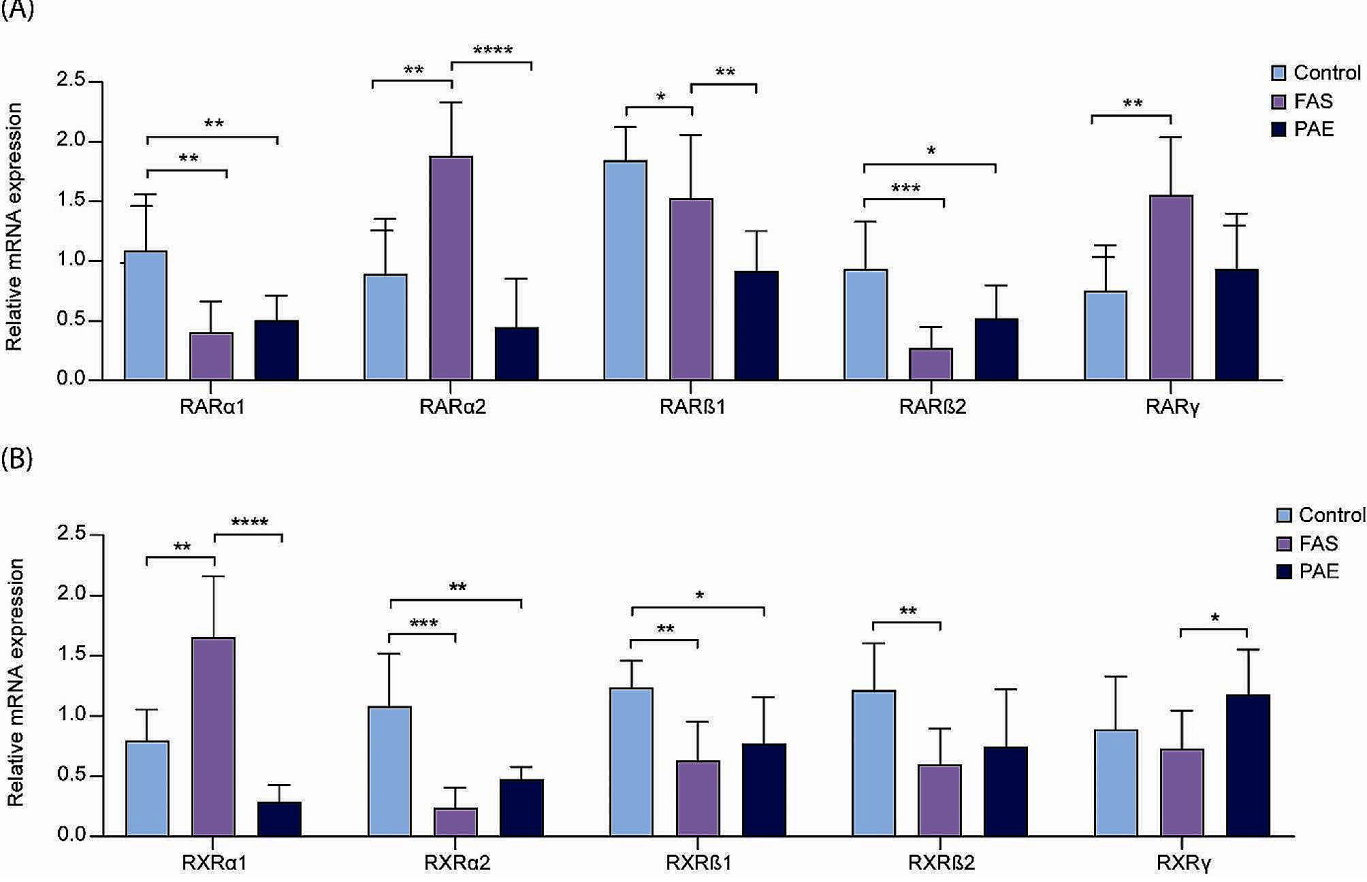
Relative expression of *RAR* (**A**) and *RXR* (**B**) by RT-qPCR in blood samples from control, FAS and PAE patients. Expression levels were normalized using GAPDH and actin as reference genes. Differences of relative RNA expression between studied groups was tested by one-way analysis of variance (ANOVA) using GraphPad Prism8 Software. * *p* < 0.05, **p < 0.01, ***p < 0.001

A significant increase in the expression of *RARα2* (*p* < 0.0116) and *RARγ* (*p* < 0.0132) was observed in the FAS group compared to the control group (Fig. 2A). *RARα2* was also significantly increased compared to the PAE group. However, the opposite was observed for *RARα1* (*p* < 0.0012), *RARβ2* (*p* < 0.0016), *RXRα2* (*p* < 0.0004), *RXRβ1* (*p* < 0.0039) and *RXRβ2* (*p* < 0.0063), where their expression was significantly decreased in FAS compared to the control group (Fig. 2A and B).

In PAE, the relative expression of *RARα1*, *RXRα2*, *RXRβ1* and *RXRβ2* was significantly lower compared to the control group. There was also a significant decrease in *RARα2* and *RXRα1* expression in the PAE group compared to FAS. The opposite was true for *RXRγ*, where a significant increase in expression was observed in PAE subjects.

### Hardy-Weinberg

The study found that most groups maintained Hardy-Weinberg equilibrium, as the p-value for each analyzed genotype was greater than 0.05, indicating stable allele segregation across generations. However, deviations from this equilibrium were observed in the FAS group with specific genetic variations in ADH1B (rs1229954), ADH1C (rs1693482), and ADH4 (rs11266773 and rs11266771). The control group, characterized by variations in ADH1C (rs6998 and rs1693482), and ADH4 (rs11266773, rs1860759, and rs11266771), also deviated from the equilibrium. Finally, the PAE group, with ADH4 variations (rs11266773 and rs11266771) showed a deviation from the equilibrium (Additional file 6).

**Fig. 3:**
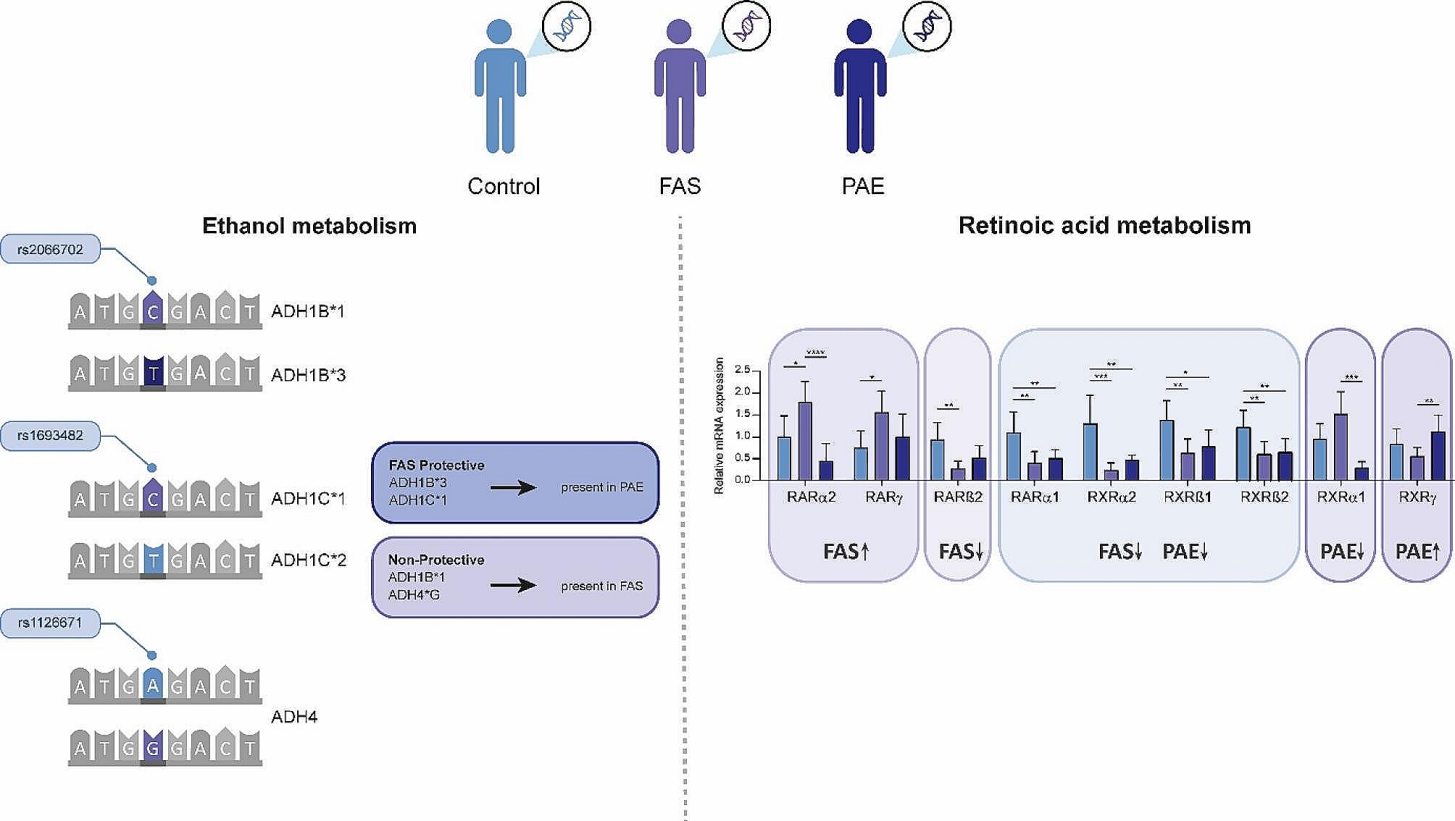
Graphical summary of the most relevant results of this study

To conclude this section, we have summarised the main findings of this study, which can be seen in Fig. 3.

## Discussion

Understanding of alcohol metabolism, its regulation and the polymorphisms of alcohol-metabolizing enzymes plays a crucial role in FAS research. Several determinants, including age, gender, ethnicity, or race, contribute to the occurrence of certain SNPs that may alter the catalytic function of hepatic, neuronal, and fetal enzymes [48–53].

The presence of SNPs in the enzymes ADH, ALDH, and CYP2E1 – the main enzymes responsible for alcohol metabolism - not only affects the way alcohol is processed, but also retinoic acid metabolism, which plays a crucial role during embryonic development. For that, many of these genetic variants were analyzed in children diagnosed with FAS, PAE and control subjects to identify specific SNPs related to alcohol metabolism.

Studies of ADH1B in European pregnant women have shown that the A allelic variant (rs1229984) is associated with reduced alcohol consumption, before and during pregnancy [54]. This is attributed to the ADH1B*2 variant, which has a higher affinity and faster metabolism of alcohol to acetaldehyde. As a result, individuals with the ADH1B*2 variant may experience a more rapid onset of alcohol-induced intoxication, leading them to stop drinking earlier than those with the ADH1B*1 isoform. ADH1B*1 is the less active form of ADH1B, that protects against a rapid increase of acetaldehyde in the blood, contributing to an increased susceptibility to alcoholism [55]. Previous ADH1B polymorphism studies have shown that the frequency of ADH1B*1 is significantly higher in men with type II alcoholism (primarily genetic, that starts during adolescence) than in men with type I alcoholism and in healthy men [56]. Rapid alcohol intoxication is also possible in people with the ADH1B*3 allele, which has higher activity than ADH1B*1. Some studies have associated the T allele of this SNP (rs2066702) in ADH1B with protection against alcohol-induced toxicity [23, 57, 58]. The increased enzymatic activity of ADH1B*2 and ADH1B*3 may protect against alcoholism, and consequently against FAS in the offspring.

There are studies that found an increased risk for alcohol-related birth defects associated with the ADH1B*1 homozygous genotype, the genotype mainly found in our FAS population. McCarver et al. found an association between maternal or infant ADH1B*1 homozygosity and the Mental Development Index (MDI) scores of those infants whose mothers drank alcohol during pregnancy [59]. Similarly, Arfsten et al. showed an increased odds for small for gestational age (SGA) in infants with prenatal alcohol exposure and homozygous for ADH1B*1, as well as an increased aggression, social problems and impulsivity [60]. In agreement with the previous study, Jacobson et al. found that ADH1B*1 homozygous mothers reported a higher mean frequency of drinking at conception (2.45 drinking days/week), compared to women with at least one ADH1B*3 allele (1.82 drinking days/week). In infants, PAE was associated with smaller head circumference and lower MDI scores only in those whose mothers were homozygous for ADH1B*1 [61]. Although our results are consistent with those discussed above in that we saw a higher proportion of ADH1B*1 in the FAS group, it is also important to note that these studies were conducted in non-Caucasian populations, such as ours. Likewise, as we do not have the genotype of the mothers, we cannot relate these genetic variants to the mechanisms of alcohol detoxification during pregnancy in this study.

Our results also showed that ADH1B has a slightly higher frequency of the AA genotype in SNP rs1229984 in FAS patients compared to controls, although the difference was not statistically significant. Regarding the SNP rs2066702, our study showed a significantly higher thymine allelic frequency in the PAE group compared to FAS group, meaning a significantly higher ADH1B*3 frequency.

Therefore, individuals with the ADH1B*3 allele, mainly PAE, have a higher rate of alcohol oxidation than individuals with only the ADH1B*1 allele, mainly FAS. It is noteworthy that the heterozygous genotype (CT) in ADH1B rs2066702 is far from the frequencies found in previous studies [62]. A study by McCarver et al. showed that the ADH1B*3 allele was only detected in African Americans and African populations and concluded that ADH1B*3 (rs2066702) acts as a protective factor against alcohol teratogenicity [62]. Embryonic damage occurs at ethanol concentrations in the range of 20–40 mM [62]. In this cellular context, ADH1B*1 is saturated, whereas ADH1B*3 can continue to oxidize alcohol. For this reason, the absence of the T allele has been associated with low birth weight in infants [62]. Although Viljoen et al. [63] reported the presence of the T allele in a mixed ancestry control population (3.9%), the presence of the ADH1B*3 isoform in individuals with PAE may allow ethanol to be metabolised more rapidly in the fetus, once it crosses the placenta and enters the fetal circulation, conferring protection against alcohol toxicity during fetal development.

In line with our findings in children exposed to ethanol but without FASD traits compared to FAS children, several studies have found a protective effect for genotypes containing ADH1B*3, possibly due to higher fetal oxidation [64]. Alterations in children’s facial morphology in children who do not have the ADH1B*3 allele and who are born to mothers who drank during pregnancy and do not carry this allele have even been seen through analysis of facial photographs. Specifically, a decrease in the inner canthal distance, the palpebral fissure length and the distance from the bridge of the nose to the base of the upper lip was observed [65]. Our study revealed a significant increase in the frequency of the T allele in the SNP rs2066702 in ADH1B, codifying for ADH1B*3 in the group prenatally exposed to alcohol (0.11) compared to the FAS group (0.01), corroborating its importance in protecting against FAS features.

The available data highlight the importance of the ADH1C enzyme in FAS. A study conducted by Molotkov et al. in 2002 suggested that the ADH1C enzyme plays a critical role in RA synthesis during development [66]. This study showed that ADH1C null mice produce less RA in vivo, resulting in growth failure and postnatal lethality, particularly under conditions of vitamin A deficiency. Based on these findings, the researchers proposed that ADH1C may function as a retinol oxidizing enzyme during development. In our study, the ADH1C rs1693482 polymorphism showed a higher frequency of the CC genotype in PAE than in controls. The C allele encodes ADH1C*1, which together with ADH1B*2 and ADH1B*3 has the highest ethanol oxidation rates and is therefore thought to promote acetaldehyde accumulation [67]. Tolstrup et al. [68] analysed the genotype of 9080 white men and women from the general population and found an odds ratio for heavy drinking of 1.4 (95% CI:1.1–1.8) in men with the ADH1C*1/2 genotype compared to the ADH1C*1/1 genotype. Therefore, individuals with ADH1C rapid alcohol metabolism had a lower risk of heavy and excessive drinking [69]. Our results suggest that children prenatally exposed to alcohol with no FASD diagnosis had a higher frequency of ADH1B*3 and ADH1C*1 alleles, which are associated not only with a protective factor against alcoholism, but also with a lower teratogenic effect, as alcohol is broken down more rapidly once it enters the fetal circulation [57].

Compared to the A allele, the G allele of the ADH4 (rs1126671) SNP reduces the thermostability and its binding capacity to ethanol [70]. Our results showed a significantly higher frequency of the GG genotype for rs1126671 in FAS patients, which would increase sensitivity to maternal alcohol in early development due to its weak binding to the teratogen [71]. On the other hand, if the mother had the G allele of the ADH4 rs1126671 SNP, she would have an ADH4 with a lower capacity to bind alcohol, so there would be a greater transfer to the fetus as it would not achieve effective alcohol oxidation. It is also known that in the human ADH family, ADH4 is the most efficient form for retinol oxidation compared to other members [72]. Therefore, if the mother has a genetic profile that modifies ADH4 activity could keep down the RA pathway, which is related to neuronal plasticity, embryogenesis [36] and oxidative stress response, playing a critical role in FAS [47]. However, as we do not have the maternal genetic information, we cannot confirm that these mothers in the FAS group had this genotype. We can also think that this variant of ADH4 in FAS children could directly affect the production of RA during embryogenesis, since the expression of ADH4 coincides spatiotemporally with the synthesis of RA in mouse embryos and that treatment with an ethanol reduces its synthesis in 7.5 day old embryos [73]. However, it is known that in the fetus the enzyme RDH10 is mainly responsible for the oxidation of retinol to retinal [33] and that ADH4 only plays a role in RA metabolism during postnatal growth and development. It would therefore be interesting to perform future studies on the expression and genotyping of RDH10 in a FAS population.

It is important to note that Saloner et al. found a significant interaction effect between alcohol use disorder and ADH4 (rs1126671) GG genotype for working memory, and at a trend level significance for executive function, in adults with co-occurring neurobiological stress conditions, such as a viral infection [70]. Individuals who were neurobiologically stressed showed dysfunction in cognitive areas of the prefrontal cortex, as is the case in children with FAS [74]. The study is consistent with our findings, as we observed a significant difference in the GG genotype of ADH4 (rs1126671) between the control and FAS groups, which represent a population with clear neurobiological stress [75, 76]. The ADH4 G allele (rs1126671), which is mainly expressed in the liver reduces the binding capacity of the enzyme to alcohol [36]. This increases its bioavailability, allowing ethanol molecules to travel to other areas such as the brain, where they can cause neurodegeneration.

In addition, our results indicated a high expression of the ALDH2 enzyme in the FAS population compared to the control and PAE groups. As ALDH2 is the enzyme responsible for the conversion of acetaldehyde to acetic acid, a higher expression of the ALDH2 in the FAS population could indicate a lower amount of acetaldehyde in the blood after alcohol exposure. Increased ALDH2 expression, together with an ADH4 variant with lower binding affinity for alcohol, may represent risk factors for alcoholism in an adult FAS population [77, 78].

CYP2E1, one of the two main enzymes that catalyse ethanol oxidation in the liver, plays an important role in the initial stages of alcohol metabolism and its activity produces high levels of ROS, generating several disorders in adult and fetal tissues such as liver and brain [79, 80] Genetic studies have investigated this enzyme in mothers and their offspring with FASD as the low metabolic activity of fetal enzymes shifts the burden of ethanol elimination to the maternal metabolism [80, 81]. Like us, the recent study by Kukowka et al. also found no statistically significant differences between FAS and control groups and the genetic polymorphisms CYP2E1, although the study does not specify the ethnicity studied, they may be Caucasian children as the study was conducted in Poland [80]. Although our results showed no significant differences in polymorphisms in this gene, we do not want to overlook the role of CYP2E1 in alcohol metabolism and its contribution to oxidative stress, further research is needed on the complex relationship between CYP2E1 polymorphisms, alcohol metabolism and oxidative stress.

This study also examined the expression of the nuclear receptors RAR and RXR, which are directly related to retinoic acid metabolism. As mentioned above, there is a competition between the RA biosynthesis and ethanol oxidation, due to their biochemical similarity [82]. Both pathways involve the production of the aldehyde intermediates retinaldehyde and acetaldehyde, which are ultimately substrates of the ALDH enzyme. Specifically, previous studies in *Xenopus* embryos demonstrated that ethanol can compete for retinaldehyde dehydrogenase during gastrulation and decrease RA levels. In contrast, the overexpression of the enzyme rescued the developmental malformations induced by the teratogen [83]. It is also important to emphasise the significance of early alcohol exposure during gastrulation, particularly between weeks 3 and 8 of gestation when the embryo is most vulnerable [84]. During this critical period, maternal detoxifying enzymes play a key role, as embryonic enzymes are not yet expressed [85]. Consequently, the characteristic facial features of FAS are often attributed to this early ethanol exposure [86, 87]. This underscores the importance of considering the embryonic susceptibility to alcohol’s effects.

Our observations revealed variations in the expression pattern within the RA pathway, suggesting a potential link between ethanol, FAS and RA. Oliveira et al. have previously shown in zebrafish embryos that any exogenous substance capable of interfering with the RAR/RXR regulatory system, such as alcohol, is a potential threat to early embryonic development by affecting the transcription of developmental genes [88].

Alcohol-exposed children have been shown to have impaired mRNA levels of the transcription factors RAR/RXR, whose signalling affects neuronal proliferation, differentiation, and physiology in the fetal brain [89–91]. RAR/RXR is also involved in the protection against oxidative stress through the binding of vitamin A to RARα, RARβ, and RXR [92]. Reduced expression of this pathway would therefore be associated with increased oxidative stress, a well-characterized part of FAS aetiology [93], which affects not only neurodevelopment but also the proper functioning of other organs such as the heart [94, 95]. Impaired RAR/RXR signalling accelerates the generation of ROS, which activates the c-Jun N-terminal kinase (JNK) signalling pathway, which is highly active in the CNS, leading to cardiomyocyte apoptosis and further RAR/RXR repression [96].

Our results showed a significant decrease in *RARα1* and *RARβ2*, in FAS patients compared to controls. RARβ2 is required for AR-induced axonal regeneration of sensory neurons and contributes to axon outgrowth by inducing axonal regeneration programmes within injured neurons [97, 98]. It has also been observed that a decrease in RARα1 expression is associated with problems during fetal morphogenesis [99]. Interestingly, our results showed a significant reduction of *RARβ2* and *RXRβ1/2* in FAS patients compared to controls, which may be related to dopaminergic dysfunction in this population, impairing learning, and action execution. Indeed, previous studies in *RARβ-RXRβ* double mutant mice have shown dysfunction of the mesolimbic dopamine signalling pathway by decreasing the levels of dopamine D1R and D2R receptor transcripts [100].

Other data to consider is the significantly increased expression of *RARα2* and *RARγ* in FAS compared to controls. Although the function of RARα2 is not well understood, previous studies have associated overexpression of this gene with reduced survival in certain myelomas due to its interaction with the JAK/STAT and MEK/ERK pathways [101]. In addition, loss of function experiments with RARα2 and RARγ by nutritional deprivation of RA have shown that they are essential for avian embryo survival and for specific events in cardiac morphogenesis [102]. Studies in mice have even shown that deregulation of RARα and RARγ promotes skin inflammation and damage to skin barrier properties [103]. In our case, we found an overexpression of *RARα2* and *RARγ* in FAS. This finding has not been previously reported in this population and warrants further investigation.

Our results also showed a significant reduction in *RXRα2*, *RXRβ1* and *RXRβ2* expression in FAS and PAE children compared to controls. RXR plays a critical role in lipid and glucose metabolism in neurons and abnormal signalling affects neuronal stress and neuroinflammatory networks. Reduced expression of these *RXR* genes has been identified in a mouse model of Alzheimer’s disease [104], while their overexpression attenuated neuronal loss and improved cognition and synaptic integrity [105]. In addition, these *RXR* genes are also involved in the modulation of the unfolded protein response and the endoplasmic reticulum stress pathways, associated with fetal brain damage in the presence of alcohol during organogenesis [106]. All these results suggest a decrease in the expression of key factors related to the protection against oxidative stress damage, neuronal stress, cardioprotection and neuronal regeneration in both FAS and PAE children. As for *RXRγ*, a significant increase in PAE could imply a higher degree of protection against oxidative stress and apoptosis, compared to the FAS population, since its inhibition generates an increase in ROS and COX-2 [107, 108].

The main limitation of this study is the size of the population analysed; it is necessary to increase the number of individuals genotyped to improve the statistical power to identify specific SNPs that cannot be discriminated in this study. We also found that certain genotypes were not in Hardy-Weinberg equilibrium, probably due to the small sample size mentioned above, although we believe that a larger sample size could resolve this situation. Likewise, it would also have been interesting to know the mother’s genotype in order to take into account her metabolic and genetic environment and to determine the origin of each allele in the child. Unfortunately, it was not possible to obtain the maternal DNA for genotyping as these children came from Eastern European orphanages, where there is little or no information about the children’s parents. FASD is not as well-known as other health conditions and there are very few FASD clinics offering a full diagnostic service. Therefore, studies of children with this condition, particularly the most severe form of the disorder (FAS), are extremely useful in further understanding the key determinants of this hidden epidemic.

## Conclusions

In conclusion, our results show that children with no FASD diagnosis but prenatally exposed to alcohol have a higher frequency of the T and C alleles in the ADH1B*3 and ADH1C*1 polymorphisms, respectively, which may partially protect them from the teratogenic effects of ethanol. In addition, unlike the FAS population, they also have the ADH1B*3 (rs2066702) and ADH1C*1 (rs1693482) polymorphisms, which cause a faster accumulation of acetaldehyde in the blood when they drink alcohol, protecting them from possible alcoholism in adulthood. In the FAS population, we have observed a dominance of isoforms associated with a lower availability of RA in the fetus and a higher capacity to convert acetaldehyde to acetic acid. This results in a lower flushing effect and could increase the risk of alcoholism in adulthood. However, despite certain protective features in partially exposed individuals, our results have also shown that this population, together with FAS, has a potential imbalance in the retinoic acid pathway. Our data may also suggest the use of RAR and RXR as potential biomarkers of prenatal alcohol exposure for early diagnosis of FAS.

## Electronic supplementary material

Below is the link to the electronic supplementary material.

Supplementary Material 1

Supplementary Material 2

Supplementary Material 3

Supplementary Material 4

Supplementary Material 5

Supplementary Material 6

Supplementary Material 7

## Data Availability

Sequence data are publicly available and can be downloaded from GenBank under the accession numbers: SAMN41738628 (ADH1), SAMN41738629 (ADH1C), SAMN41738630 (ADH4), SAMN41738631(ALDH1A1), SAMN41738632 (ALDH2) and SAMN41738633 (CYP2E1). The data that support the findings of this study are publicly available from Zenodo with the identifier 10.5281/zenodo.11484200 (https://zenodo.org/records/11484200). The original gel image from Additional file 5 can be found in Additional file 7.

## Abbreviations

ADH: Alcohol Dehydrogenases
ALDH: Aldehyde Dehydrogenase
ARBD: Alcohol-Related Birth Defects
ARND: Alcohol-Related Neurodevelopmental Disorder
CNS: Central Nervous System
CYP2E1: Cytochrome P450 2E1
EEC: Eastern European Countries
FAS: Fetal Alcohol Syndrome
FASD: Fetal Alcohol Spectrum Disorders
HWE: Hardy–Weinberg Equilibrium
PAE: Prenatal Alcohol Exposure
pFAS: Partial Fetal Alcohol Syndrome
RA: Retinoic Acid
RALDH: Retinaldehyde Dehydrogenase
RAR: Retinoic Acid Receptor
ROS: Reactive Oxygen Species
RXR: Retinoid X receptor
SNPs: Single Nucleotide Polymorphisms
RAREs: Retinoic Acid Response Elements
RXRE: Retinoid X Response Element
PAE: Prenatal alcohol exposure
JNK: C-Jun N-terminal Kinase
