# Correction: Analysis of alcohol-metabolizing enzymes genetic variants and RAR/RXR expression in patients diagnosed with fetal alcohol syndrome: a case-control study

**DOI:** 10.1186/s12864-024-10766-5

**Published:** 2024-09-05

**Authors:** Melina Vieiros, Elisabet Navarro-Tapia, Anna Ramos-Triguero, Àgueda García-Meseguer, Leopoldo Martínez, Óscar García-Algar, Vicente Andreu-Fernández

**Affiliations:** 1 Grup de Recerca Infància i Entorn (GRIE), Institut d’investigacions Biomèdiques August Pi i Sunyer (IDIBAPS), Barcelona, Spain; 2 IdiPAZ - Instituto de Investigación Hospital Universitario La Paz, Madrid, Spain; 3 Department de Cirurgia i Especialitats Mèdico-Quirúrgiques, Universitat de Barcelona, Barcelona, Spain; 4 Faculty of Health Sciences, Valencian International University, Valencia, Spain; 5 Department of Pediatric Surgery, Hospital Universitario La Paz, Madrid, Spain; 6 Department of Neonatology, Hospital Clínic-Maternitat, ICGON, BCNatal, Barcelona, Spain; 7 Biosanitary Research Institute, Valencian International University, Valencia, Spain

**Correction:** ***BMC Genomics*** **25, 610 (2024)**

10.1186/s12864-024-10516-7

Following publication of the original article it was reported that Melina Vieiros was missing the following affiliation: Department de Cirurgia i Especialitats Mèdico-Quirúrgiques, Universitat de Barcelona, Barcelona, Spain.

The updated authorship information is available in this Correction article and the original article has been updated.

