# Supplementary material for: Analysis of alcohol-metabolizing enzymes genetic variants and RAR/RXR expression in patients diagnosed with fetal alcohol syndrome: a case-control study
Source: BMC Genomics. 2024 Jun 17;25:610. doi: 10.1186/s12864-024-10516-7 (PMC11184718; doi:10.1186/s12864-024-10516-7)
Supplement: Supplementary file 1 — Supplementary Material 1 [file 12864_2024_10516_MOESM1_ESM.pdf]

Additional file 1. PCR optimal temperature for melting and sequence of primers used to carry out Tetra-ARMS-PCR.

Enzyme	SNP	Allele	Tm ºC	Total length	Allele-specific length	Forward Inner sequence (5’ to 3’)	Reverse Inner sequence (5’ to 3’)	Forward Outer sequence (5’ to 3’)	Reverse Outer sequence (5’ to 3’)
ADH1B	rs1229984	A	63	640 bp	443 bp	GTGGCTGTAGGAATCTGTCA	CCACGTGGTCATCTGTGC	GAAGGGAAGGTAGAGAAGGG	GACTATGAATGCCACACTGG
		G			234 bp				
ADH1B	rs2066702	C	58	424 bp	310 bp	CTTCTTTCCTATTGCAGTATCC	CAAAACGTCAGGACGGTACA	GGGGATGGAAATAGGGTAG	GGTGACACAGTAGAATGGTTAAG
		T			155 bp				
ADH1C	rs698	A	55	765 bp	348 bp	CACTGGATGCATTAATAACAAATA	CATTTATTTTTTCAAAAGGTAAAAC	GTACTTGTGACTGCGTAAGGC	GAAGGCTGAATGACTCTGATC
		G			465 bp				
ADH1C	rs1693482	T	65	588 bp	233 bp	CATACCATGGTGTCAAGCC	CGTTTGAAGTCATCGGTCA	GACTTGAGACAGGTTTGCTTAG	CGATAACAGATTGGACAGAAGAG
		C			395 bp				
ADH4	rs1126673	G	58	434 bp	320 bp	TGTTTTATTGTTATTAACATCG *	AAGATGAGGATTGTTCGTAT *	GAAATTAGGCTTAATAAAGTGAATG	CCCACTTTCAACTTCCCAG
		A			155 bp				
ADH4	rs1042364	A	60	604 bp	426 bp	GATGCCAGGAGCAATTCA	CACATTCAATCAGATAGTATTCC	GAGGATGACGGGATAAAAG	GTCCATGTGTTCTCATCATTTAG
		G			218 bp				
ADH4	rs1800759	A	52.5	525 bp	208 bp	GCAACAAAGGAGAAAAGGA	GTTGCTTAATTCTCCAATCACTG	GATTAAACTATTGGATAGAGAAGC	GCACAAACTGAACAAAGATACAG
		C			358 bp				
CYP2E1	rs2031920	C	50	449 bp	299 bp	AGATTCATTGTTAATATAAAAGGAC *	TAATTCATAGGTTGCAATTGTA *	CTGGGTTAGAATGCAATATATAG	ATTGGATTTGTTTTACATTAGG
		T			196 bp				
CYP2E1	rs3813867	G	60	800 bp	358 bp	CTTCTTGGTTCAGGAGAGG	GCTGCACCTAACACTGCAG	GGTGAGCTGCACAGACAAG	GAGCTTTTTTGTGTGTGTGG
		C			479 bp				
CYP2E1	rs72559710	G	65	356 bp	141 bp	GTACGTGGGCTCGCAGCG	CGTGCATCACCACCATGT	CAGACGCAGCAGCCTCTTG	GACGCGGACTCACCCCTG
		A			251 bp				
CYP2E1	rs6413419	G	65	648 bp	415 bp	GCTGCGCGCCCTGCAACG	GAAGAGGATGTCGGCTATGAT	GCCATTAAACACGTGACTTG	GGGTCAACAGTGTCCTTCTTG
		A			271 bp				
CYP2E1	rs55897648	G	60	393 bp	170 bp	TCTCCTAGGGCACAGTCG	CAGAGTCCAGAGTTGGCACTAT	CTATCACAACCAGCCCTGG	GCCTCTGATCTTTCTCACCTG
		A			265 bp				
ALDH1A1	rs8187929	A	60	784 bp	323 bp	TTCCCGTTGGTTATGCTCA	CAGGCCCTATCTTCCAAAA	CTTGGCCTCTGGACCCTG	GGCTTCCTGTCTGCATTTG
		T			497 bp				

*: mismatch introduced at the penultimate position of the 3’ terminus
