# Supplementary material for: Analysis of alcohol-metabolizing enzymes genetic variants and RAR/RXR expression in patients diagnosed with fetal alcohol syndrome: a case-control study
Source: BMC Genomics. 2024 Jun 17;25:610. doi: 10.1186/s12864-024-10516-7 (PMC11184718; doi:10.1186/s12864-024-10516-7)
Supplement: Supplementary file 2 — Supplementary Material 2 [file 12864_2024_10516_MOESM2_ESM.pdf]

Additional file 2. PCR optimal temperature for melting and sequence of primers used for sequencing method.

Enzyme	SNP	Tm ºC	Length	Forward sequence (5' to 3')	Reverse sequence (5' to 3')
ADH4	rs1126671<br>rs29001219	65	330 bp	GTACATTGCCACACTGGAGTTC	GTATTTGGATATGCTCTAGGG
CYP2E1	rs6413432	68	646 bp	GTTCACAGCCTGAGTGGTG	GCTAGAGTGCAGTGGGGTG
ALDH1A1	rs1049981<br>rs11554423	65	357 bp	GGAGGTTAGACAAGATGACAAG	GGTTGACATCTTTAAAAGGG
ALDH2	rs671<br>rs769724893	65	355 bp	GACTTTGGGGCAATACAGG	GCAGGTCCTGAACTTCCAG
