# Supplementary material for: Analysis of alcohol-metabolizing enzymes genetic variants and RAR/RXR expression in patients diagnosed with fetal alcohol syndrome: a case-control study
Source: BMC Genomics. 2024 Jun 17;25:610. doi: 10.1186/s12864-024-10516-7 (PMC11184718; doi:10.1186/s12864-024-10516-7)
Supplement: Supplementary file 3 — Supplementary Material 3 [file 12864_2024_10516_MOESM3_ESM.pdf]

Additional file 3. PCR optimal temperature for melting and sequence of primers used in for expression level analysis.

Transcription factor	Isotype	Isoform	Cycles	Tm ºC	Length	Forward sequence (5' to 3')	Reverse sequence (5' to 3')
RAR	RARα	α1	33	65	460 bp	CGCTCTGACCACTCTCCAG	GCGCACCTTCTCAATGAG
		α1δDC			169 bp		
RAR	RARα	α2	33	67.5	559 bp	GTACGAGAGTGTAGAAGTGGGG	GCGCACCTTCTCAATGAG
RAR	RARβ	β1	33	61.3	67 bp	AAAGTGAGGGAGGCAAATGC	CATCTTGACTTTGGCCCAGA
RAR	RARβ	β2	33	65.8	312 bp	ATTCATGATTCGGGGCTGGG	TCAATTGATTGAGCAGTGTGCC
RAR	RARγ	γ1	33	65	601 bp	GCCTTTCGAGATGCTGAG	GCACTTGGTAGCCAGCTCAC
RXR	RXRα	α1	33	61	522 bp	GATTTCTCCACCCAGGTG	CTGCCGCTTGTCAATCAG
RXR	RXRα	α2	31	61	266 bp	GCTCACCTATGAACCCCG	CTGCCGCTTGTCAATCAG
RXR	RXRβ	β1	33	65	349 bp	GAGCTTGCTGTGGAACAGAAG	GCTCTGTCAGCACCCGATC
RXR	RXRβ	β2	33	65	348 bp	GAGCTTGCTGTGGAACAGAAG	CCTGGAGAGGGACCGATC
RXR	RXRγ	γ1	33	60	2768 bp	CAACAGGATCTCAAGCAGCA	CAGCTGGTGCCTGTCAGTAA
**Control gene**	**Isotype**	**Isoform**	**Cycles**	**Tm ºC**	**Length**	**Forward sequence (5' to 3')**	**Reverse sequence (5' to 3')**
GAPDH	-	-	29	65	87 bp	TGCACCACCAACTGCTTAGC	GGCATGGACTGTGGTCATGAG
