# Supplementary material for: Analysis of alcohol-metabolizing enzymes genetic variants and RAR/RXR expression in patients diagnosed with fetal alcohol syndrome: a case-control study
Source: BMC Genomics. 2024 Jun 17;25:610. doi: 10.1186/s12864-024-10516-7 (PMC11184718; doi:10.1186/s12864-024-10516-7)
Supplement: Supplementary file 4 — Supplementary Material 4 [file 12864_2024_10516_MOESM4_ESM.pdf]

ADDITIONAL FILE

Additional file 4. (A) Genotype and allele frequencies of ALDH1A1 (for rs8187929, rs1049981, rs11554423) and ALDH2 (rs671, rs769724893) in control, FAS and PAE children. (B) Genotype and allele frequencies of CYP2E1 (for rs2031920, rs3813867, rs72559710, rs6413432, rs6413419 and rs55897648 in control, FAS and PAE children.

(A)

**ALDH1A1**											
**SNP**			**Control**		**FAS**		**PAE**		**Statistical hypothesis testing**		
			n	Freq	n	Freq	n	Freq	Groups	χ2	*p*-value
rs8187929 (A → T)	**Genotype**	AA	28	1	34	1	9	1	Ctrl vs FAS	0	1
		AT	0	0	0	0	0	0	Ctrl vs PAE	0	1
		TT	0	0	0	0	0	0	PAE vs FAS	0	1
	**Allele**	A	56	1	68	1	18	1	Ctrl vs FAS	0	1
		T	0	0	0	0	0	0	Ctrl vs PAE	0	1
									PAE vs FAS	0	1
rs1049981 (G → A)	**Genotype**	AA	28	1	34	1	9	1	Ctrl vs FAS	0	1
		AG	0	0	0	0	0	0	Ctrl vs PAE	0	1
		GG	0	0	0	0	0	0	PAE vs FAS	0	1
	**Allele**	A	56	1	68	1	18	1	Ctrl vs FAS	0	1
		G	0	0	0	0	0	0	Ctrl vs PAE	0	1
									PAE vs FAS	0	1
rs11554423 (G → C)	**Genotype**	GG	28	1	34	1	9	1	Ctrl vs FAS	0	1
		GC	0	0	0	0	0	0	Ctrl vs PAE	0	1
		CC	0	0	0	0	0	0	PAE vs FAS	0	1
	**Allele**	G	56	1	68	1	18	1	Ctrl vs FAS	0	1
		C	0	0	0	0	0	0	Ctrl vs PAE	0	1
									PAE vs FAS	0	1
**ALDH2**											
rs671 (G → A)	**Genotype**	GG	28	1	34	1	9	1	Ctrl vs FAS	0	1
		AG	0	0	0	0	0	0	Ctrl vs PAE	0	1
		AA	0	0	0	0	0	0	PAE vs FAS	0	1
	**Allele**	G	56	1	68	1	18	1	Ctrl vs FAS	0	1
		A	0	0	0	0	0	0	Ctrl vs PAE	0	1
									PAE vs FAS	0	1
rs769724893 (G → A)	**Genotype**	GG	28	1	34	1	9	1	Ctrl vs FAS	0	1
		AG	0	0	0	0	0	0	Ctrl vs PAE	0	1
		AA	0	0	0	0	0	0	PAE vs FAS	0	1
	**Allele**	G	56	1	68	1	18	1	Ctrl vs FAS	0	1
		A	0	0	0	0	0	0	Ctrl vs PAE	0	1
									PAE vs FAS	0	1

(B)

CYP2E1	SNP		Control n	Control Freq	FAS n	FAS Freq	PAE n	PAE Freq	Groups	χ2	*p*-value
rs2031920 (C → T)	**Genotype**	CC	28	1	34	1	9	1	Ctrl vs FAS	0	1
		CT	0	0	0	0	0	0	Ctrl vs PAE	0	1
		TT	0	0	0	0	0	0	PAE vs FAS	0	1
	**Allele**	C	56	1	68	1	18	1	Ctrl vs FAS	0	1
		T	0	0	0	0	0	0	Ctrl vs PAE	0	1
									PAE vs FAS	0	1
rs3813867 (G → C)	**Genotype**	GG	28	1	34	1	9	1	Ctrl vs FAS	0	1
		CG	0	0	0	0	0	0	Ctrl vs PAE	0	1
		CC	0	0	0	0	0	0	PAE vs FAS	0	1
	**Allele**	G	56	1	68	1	18	1	Ctrl vs FAS	0	1
		C	0	0	0	0	0	0	Ctrl vs PAE	0	1
									PAE vs FAS	0	1
rs72559710 (G → A)	**Genotype**	GG	27	0.96	34	1	9	1	Ctrl vs FAS	1.23	0.54
		GC	1	0.04	0	0	0	0	Ctrl vs PAE	0.33	0.85
		CC	0	0	0	0	0	0	PAE vs FAS	0	1
	**Allele**	G	55	0.98	68	1	18	1	Ctrl vs FAS	1.22	0.27
		C	1	0.02	0	0	0	0	Ctrl vs PAE	0.33	0.57
									PAE vs FAS	0	1
rs6413432 (A → T)	**Genotype**	TT	28	1	32	0.94	9	1	Ctrl vs FAS	1.70	0.43
		AT	0	0	2	0.06	0	0	Ctrl vs PAE	0	1
		AA	0	0	0	0	0	0	PAE vs FAS	0.55	0.76
	**Allele**	T	56	1	66	0.97	18	1	Ctrl vs FAS	1.67	0.20
		A	0	0	2	0.03	0	0	Ctrl vs PAE	0	1
									PAE vs FAS	0.54	0.46
rs6413419 (G → C)	**Genotype**	GG	22	1	32	1	9	1	Ctrl vs FAS	3.30	0.19
		AG	6	0	2	0	0	0	Ctrl vs PAE	0.37	0.83
		AA	0	0	0	0	0	0	PAE vs FAS	0.55	0.76
	**Allele**	G	50	1	66	1	18	1	Ctrl vs FAS	3.07	0.08
		A	6	0	2	0	0	0	Ctrl vs PAE	0.17	0.68
									PAE vs FAS	0.54	0.46
rs55897648 (A → G)	**Genotype**	GG	28	1	34	1	9	1	Ctrl vs FAS	0	1
		AG	0	0	0	0	0	0	Ctrl vs PAE	0	1
		AA	0	0	0	0	0	0	PAE vs FAS	0	1
	**Allele**	G	56	1	68	1	18	1	Ctrl vs FAS	0	1
		A	0	0	0	0	0	0	Ctrl vs PAE	0	1
									PAE vs FAS	0	1

Abbreviations: FAS: Fetal Alcohol Syndrome; PAE: Prenatal Ethanol Exposure. * Indicates statistical significance (*p*-value <0.05). χ2: Chi-square test.
