# Supplementary material for: Analysis of alcohol-metabolizing enzymes genetic variants and RAR/RXR expression in patients diagnosed with fetal alcohol syndrome: a case-control study
Source: BMC Genomics. 2024 Jun 17;25:610. doi: 10.1186/s12864-024-10516-7 (PMC11184718; doi:10.1186/s12864-024-10516-7)
Supplement: Supplementary file 5 — Supplementary Material 5 [file 12864_2024_10516_MOESM5_ESM.pdf]

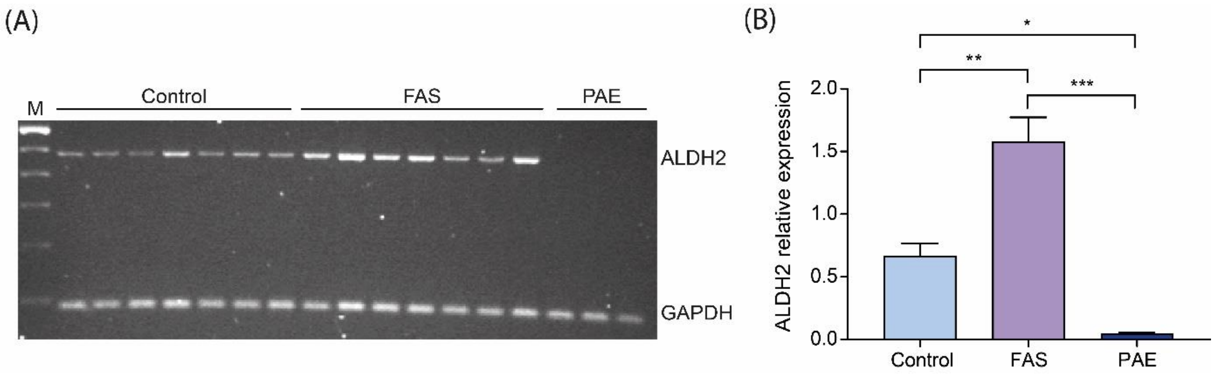

Additional file 5. Gene expression analysis of ALDH2 isoform 1 using semi-quantitative RT-PCR. Human blood samples from EEC were used to perform RT-PCR expression experiments. (A) Human ALDH2 isoform 1 mRNA expression (Lanes 1-7: control group. Lanes 8-14: FAS samples. Lanes 15-17: PAE). (B) Relative expression levels of ALDH2 mRNA in 10 Controls, 10 FAS and 3 PAE samples. Fold Change was calculated using relative values of GAPDH control mRNA for each sample analysed. T-test were used to compare mRNA expression among groups. *p<0.05, **p<0.01, ***p< 0.001
