# Supplementary material for: Analysis of alcohol-metabolizing enzymes genetic variants and RAR/RXR expression in patients diagnosed with fetal alcohol syndrome: a case-control study
Source: BMC Genomics. 2024 Jun 17;25:610. doi: 10.1186/s12864-024-10516-7 (PMC11184718; doi:10.1186/s12864-024-10516-7)
Supplement: Supplementary file 6 — Supplementary Material 6 [file 12864_2024_10516_MOESM6_ESM.pdf]

Additional file 6. Evaluation if the population is in Hardy-Weinberg equilibrium.

Gene	SNP	HWE Groups	$\chi2$	*p*-value
ADH1B	rs1229984 (G →A)	Ctrl	3.26	0.07
		FAS	24.0	9.47E-07*
		PAE	0.03	0.86
	rs2066702 (C → T)	Ctrl	0.17	0.68
		FAS	0.007	0.93
		PAE	0.14	0.7
ADH1C (γ)	rs698 (A → G)	Ctrl	4.48	0.03*
		FAS	0.02	0.90
		PAE	0.09	0.76
	rs1693482 (C → T)	Ctrl	20.6	5.77E-06*
		FAS	10.3	0.001*
		PAE	0.25	0.61
ADH4 (π)	rs1126673 (A → G)	Ctrl	10.1	0.001*
		FAS	8.6	0.003*
		PAE	5.76	0.02*
	rs1042364 (A→ G)	Ctrl	0.78	0.38
		FAS	1.41	0.23
		PAE	0.03	0.86
	rs1800759 (A → C)	Ctrl	7.50	0.006*
		FAS	0.86	0.35
		PAE	3.64	0.06
	rs1126671 (A → G)	Ctrl	5.32	0.02*
		FAS	5.32	0.02*
		PAE	0.24	0.62
	rs29001219 (A → G)	Ctrl	0	1
		FAS	0	1
		PAE	0	1

Gene	SNP	HWE Groups	$\chi2$	*p*-value
ALDH1	rs8187929 (A → T)	Ctrl	0	1
		FAS	0	1
		PAE	0	1
	rs1049981 (G → A)	Ctrl	0	1
		FAS	0	1
		PAE	0	1
	rs11554423 (G → C)	Ctrl	0	1
		FAS	0	1
		PAE	0	1
ALDH2	rs671 (G → A)	Ctrl	0	1
		FAS	0	1
		PAE	0	1
	rs769724893 (G → A)	Ctrl	0	1
		FAS	0	1
		PAE	0	1

Gene	SNP	HWE Groups	$\chi2$	*p*-value
CYP2E1	rs2031920 (C → T)	Ctrl	0	1
		FAS	0	1
		PAE	0	1
	rs3813867 (G → C)	Ctrl	0	1
		FAS	0	1
		PAE	0	1
	rs72559710 (G → A)	Ctrl	0.01	0.92
		FAS	0	1
		PAE	0	1
	rs6413432 (A → T)	Ctrl	0	1
		FAS	0.03	0.86
		PAE	0	1
	rs6413419 (G → C)	Ctrl	0.40	0.52
		FAS	0.03	0.86
		PAE	0	1
	rs55897648 (A → G)	Ctrl	0	1
		FAS	0	1
		PAE	0	1

HWE ($\chi2$) represents the value of the analysis of variances, HWE (*p*) indicates the p-value. The asterisk (*) represents a significance level or p-value ≤ 0.05.
