# Supplementary material for: Analysis of alcohol-metabolizing enzymes genetic variants and RAR/RXR expression in patients diagnosed with fetal alcohol syndrome: a case-control study
Source: BMC Genomics. 2024 Jun 17;25:610. doi: 10.1186/s12864-024-10516-7 (PMC11184718; doi:10.1186/s12864-024-10516-7)
Supplement: Supplementary file 7 — Supplementary Material 7 [file 12864_2024_10516_MOESM7_ESM.pdf]

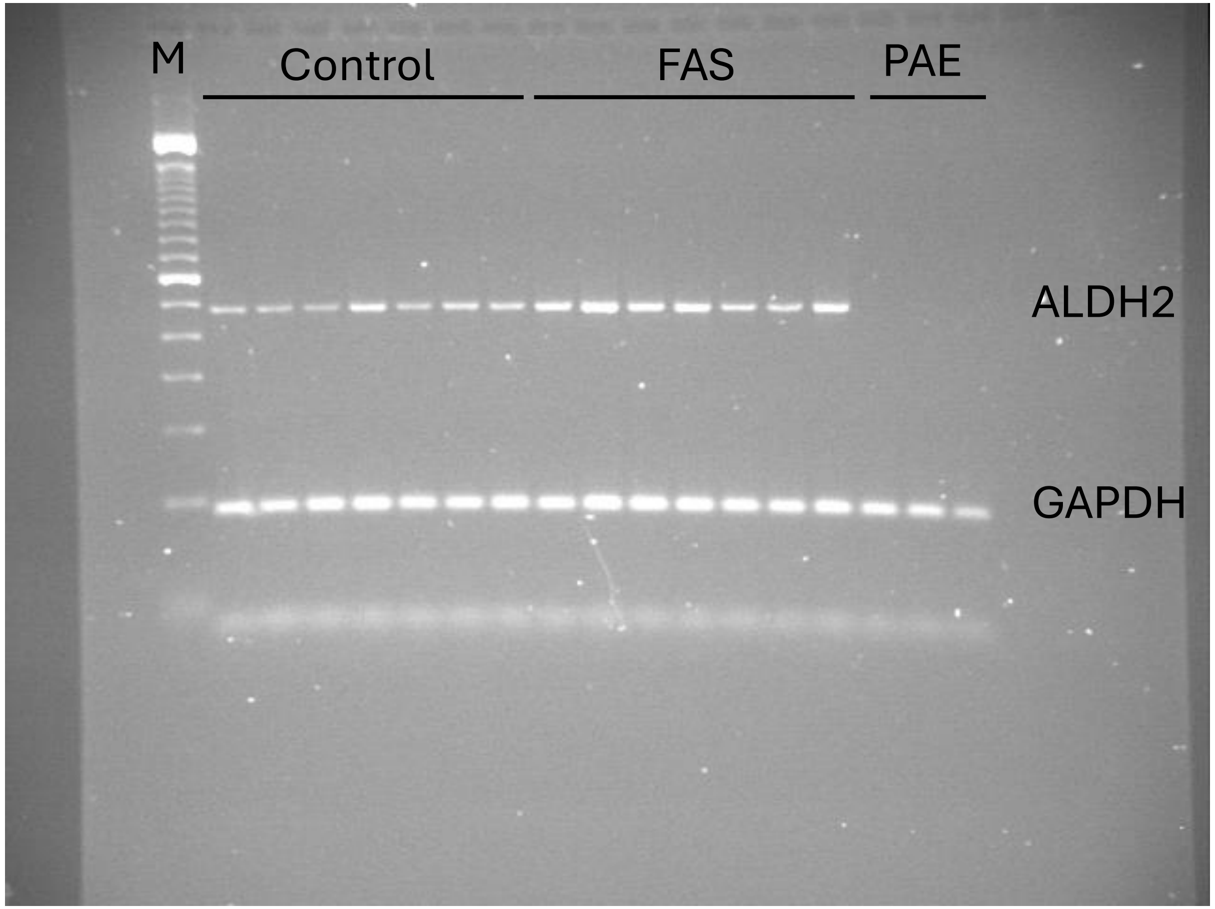

Gene expression analysis of ALDH2 isoform 1 using semi-quantitative RT-PCR. Human blood samples from EEC were used to perform RT-PCR expression experiments.
Lanes 1-7: control group. Lanes 8-14: FAS samples. Lanes 15-17: PAE.
